# Phosphorylation-mediated regulation of integrin-linked kinase 5 by purinoreceptor P2K2

**DOI:** 10.1080/15592324.2023.2261743

**Published:** 2023-12-17

**Authors:** Daewon Kim, Gary Stacey

**Affiliations:** Division of Plant Science and Technology, C.S. Bond Life Science Center, University of Missouri, Columbia, MO, USA

**Keywords:** Extracellular ATP, purinergic signaling, P2K2, purinoreceptor, Integrin-linked kinase 5 (ILK5), danger-associated molecular patterns (DAMPs)

## Abstract

Extracellular ATP (eATP) in plants plays a crucial role as a ligand for purinoreceptors, mediating purinergic signaling and regulating diverse biological functions, including responses to abiotic and biotic stresses. DORN1/P2K1 (LecRK I.9) was the first identified plant purinoreceptor. P2K2 (LecRK I.5) was subsequently identified as an additional plant purinoreceptor and shown to directly interact with P2K1. Recently, we reported that P2K1 interacts with Integrin-linked kinase 5 (ILK5), a Raf-like MAPKKK protein, and phosphorylates ILK5 to regulate purinergic signaling in relation to plant innate immunity. Here, we report that P2K2 also interacts with the ILK5 protein *in planta*. Furthermore, we demonstrate that P2K2 phosphorylates ILK5 in the presence of [γ-32P] ATP, similar to P2K1. However, unlike P2K1, P2K2 exhibits strong phosphorylation even when the Serine 192 residue of ILK5 is mutated to Alanine (ILK5^S192A^), suggesting the possibility of phosphorylation of other residues to fully regulate ILK5 protein function.

## Introduction

ATP is utilized as a vital intracellular energy carrier and is indispensable for many cellular processes in all living organisms. However, under conditions of tissue damage or various biotic and abiotic stresses (e.g., *Pseudomonas syringae* infection or high salinity), ATP is released into the extracellular compartment.^1–4^ This released extracellular ATP (eATP) functions as a danger-associated molecular pattern (DAMP) signaling molecule in eukaryotes.^5,6^ The process of perceiving and transmitting signals through eATP is referred to as purinergic signaling, and the receptors directly involved in this process are called purinoreceptors.^5,7^ In animals, a wide range of purinoreceptors have been identified, including P2X ligand-gated ion channels and P2Y G protein-coupled receptors, which play roles in various biological processes such as tumor recognition, inflammation, neurotransmission and cell death.^8,9^ While purinergic signaling in mammals has been extensively studied and, indeed, underpins a multibillion dollar pharmaceutical market, comparatively little is known about purinergic signaling in plants.

The first purinoreceptor in plants, initially designated as DORN1 (DOes not Respond to Nucleotide 1), was identified in 2014 using an EMS-based forward genetic mutant screen.^10^ Subsequently, DORN1 was renamed P2K1 to align with the nomenclature of animal P2-type receptors and to indicate its active kinase nature (K). P2K1 belongs to the lectin receptor-like kinase protein family (LecRK I.9) and localizes to the plasma membrane.^10^ P2K1 consists of an N-terminal, extracellular ATP binding domain, a transmembrane domain, and an intracellular, C-terminal serine/threonine kinase domain.^6,10^ A number of published reports have identified downstream targets of P2K1 kinase activity, implicating purinergic signaling in both biotic and abiotic plant stress responses.^2,11–14^ These pathways encompass processes such as cytosolic calcium influx, reactive oxygen species (ROS) production, and Mitogen-Activated Protein Kinase (MAPK) phosphorylation.^2,12,14^ Recent investigations have shed light on the regulatory mechanisms governing P2K1 activity. For example, S-acylation was shown to influence P2K1 temporal dynamics through effects on auto-phosphorylation and proteolysis.^11^ Furthermore, P2K1 was shown to directly phosphorylate mevalonate kinase, exerting an impact on the synthesis of secondary metabolites and hormonal pathways in response to eATP.^13^

## Results and discussion

Previously, P2K2 (LecRK I.5) was shown to dimerize, interact with P2K1, and play a crucial role in regulating innate immunity in plants.^7^ Notably, *p2k2* knock-out mutant plants display phenotypic similarities to *p2k1* mutants. For example, they show reduced phosphorylation of MPK3/6 upon exposure to ATP compared to the wild-type Col-0 plants.^7,10^ Both *p2k1* and *p2k2* mutant plants are also defective in generating a systemic ROS response upon addition of ATP.^3^ However, at present, we know little regarding the downstream targets of P2K2 kinase activity. In this study, we investigated the interaction between P2K2 and the ILK5 Raf-like MAPKKK protein and demonstrate that ILK5 is a direct phosphorylation target of P2K2.^14^

**Figure 1.**
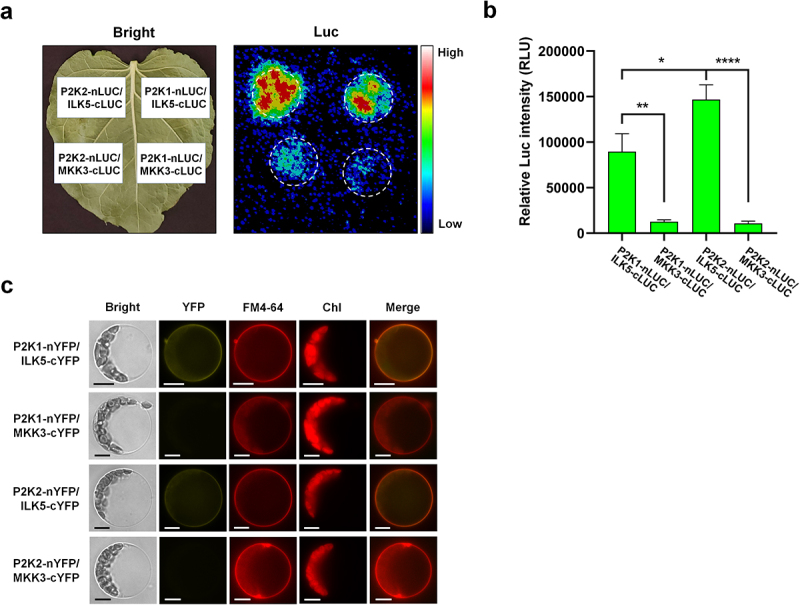
P2K2 interacts with ILK5 *in planta*.

To validate the protein-protein interaction between P2K2 and ILK5, a firefly split-luciferase complementation imaging (LCI) assay was performed with P2K1 and Mitogen-Activated Protein Kinase Kinase 3 (MKK3)^14^ as a positive and a negative control, respectively. *Agrobacterium tumefaciens* strain *GV3101*, containing the respective construct, was co-infiltrated into 4 week-old *N. benthamiana* leaves using a needleless syringe. After three days infiltration, protein interaction was detected using a low light capture system by spraying the leaf’s underside with a solution containing D-luciferin to capture luminescence light. The results revealed that P2K2, similar to P2K1, interacts with ILK5 (Figure 1a). Images were captured and luciferase signal intensities were quantified using the C-vision/Im32 software. Finally, the data were analyzed using the GraphPad Prism 8 software. As shown in Figure 1, P2K2 showed a strong signal, indicating a significant level of interaction with the ILK5 protein compared to P2K1 (Figure 1b).

(a) Investigation of P2K2-ILK5 protein-protein interaction using firefly split-luciferase complementation imaging (LCI) assay. The *GV3101* agrobacterium containing *P2K2-nLUC* and *ILK5-cLUC* constructs was co-infiltrated into *N. benthamiana* leaves for the LCI assay. The luminescence light was monitored using a low-light imaging CCD camera (Photek; Photek, Ltd.) after 3 days of infiltration, and images were captured. Dotted circles indicate the areas where *GV3101* agrobacterium was infiltrated into *N. benthamiana* leaves. P2K1-nLUC and MKK3-cLUC protein were used as a positive or a negative control, respectively. (b) Quantification of P2K2-ILK5 interaction signal intensities. The interaction between P2K2 and ILK5 was monitored, followed by image capture and quantification of the luciferase signal intensities using the C-vision/Im32 program. The data were then analyzed using the GraphPad Prism 8 software. Data shown as mean ± SEM, *n* = 7 (biological replicates), ****p<.0001, ***p<.001, **p<.01, *p<.05, *p*-value indicates significance relative to MKK3-cLUC and was determined by unpaired two-tailed Student’s *t*-test. P2K1-ILK5 was used as a positive control. (c) P2K2-ILK5 interaction was demonstrated using the biomolecular fluorescence complementation (BiFC) assay in Arabidopsis protoplasts. The specified constructs were transiently transfected into Arabidopsis protoplasts and subsequently incubated in darkness for 24 hours. The YFP fluorescence was monitored using a Zeiss Axiovert 200 M inverted Microscope with ORCA-ER camera. FM4-64 was used as a plasma membrane marker. Chl indicates the autofluorescence signal of chlorophyll. Merge represents the combined image of YFP and FM4-64. P2K1-nYFP and MKK3-cYFP were employed as a positive and negative control, respectively. Scale bars = 10 *μ*m. The experiments were repeated two times (biological replicates) with similar results.

Previously, it was reported that P2K1 and ILK5 interact at the plasma membrane.^14^ Therefore, to confirm the protein-protein interaction between P2K2 and ILK5 at the plasma membrane, a biomolecular fluorescence complementation (BiFC) assay was performed. Protoplasts were isolated from 3-week-old Arabidopsis Col-0, and each construct DNA was transformed using the PEG method.^15,16^ After transformation, the protoplasts were incubated for 24 hours, followed by the observation of YFP fluorescence using a fluorescence microscope equipped with a YFP filter. As expected, the YFP fluorescence signal was observed at the plasma membrane of the protoplasts. FM4–64 was used as a plasma membrane marker (Figure 1c).

**Figure 2.**
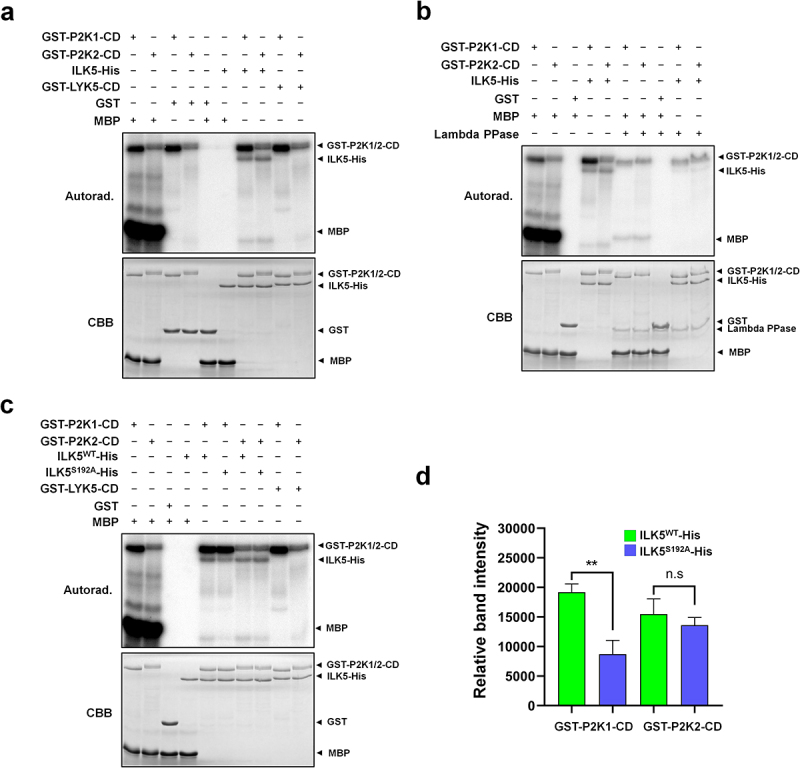
P2K2 directly phosphorylates ILK5 *in vitro*.

To verify whether P2K2 phosphorylates ILK5, GST-P2K1, GST-P2K2, and ILK5-His tagged recombinant proteins were purified using affinity chromatography (Supplemental Figure S1). *In vitro* kinase assays were conducted with a reaction buffer in the presence of [γ-32P] ATP. The results indicated that P2K2, like P2K1, strongly phosphorylates ILK5 (Figure 2a). To confirm that these signals were due to phosphorylation, lambda protein phosphatase (PPase) treatment was performed, which showed a significant reduction in ILK5 transphosphorylation (Figure 2b). Based on previous reports, P2K1 phosphorylates Ser192 of ILK5, and Ser192 plays a crucial role in plant immunity.^14^ Therefore, we conducted an *in vitro* kinase assay with ILK5^S192A^ mutant protein and P2K2 in the presence of [γ-32P] ATP. The results showed that ILK5^S192A^ exhibited significantly reduced phosphorylation by P2K1 compared to the wild-type. In contrast, the mutant protein was strongly phosphorylated by P2K2 (Figure 2c, d; Supplemental Figure S2). As per the NetPhos 3.1a software, it is possible to predict phosphorylation for several serine, threonine, and tyrosine residues within the ILK5 protein, including S192 (Supplemental Figure S3). Additionally, according to the Plant PTM Viewer (version 2.0),^17^ experimentally confirmed phosphorylation has been observed in ILK5, not only at S192 but also at residues S17, S27, S30, and S453 (Supplemental Figure S3 and Supplemental Table S1). The data suggest the potential for ILK5 phosphorylation at multiple residues in response to purinergic signaling, either through the action of P2K1 and/or P2K2.

(a) GST-P2K2-CD phosphorylates ILK5-His protein but not GST-LYK5-CD protein. In an *in vitro* kinase assay, the bacterial recombinant ILK5-His protein was subjected to incubation with either GST-P2K1 cytosolic domain (GST-P2K1-CD) or GST-P2K2 cytosolic domain (GST-P2K2-CD). (b) ILK5 phosphorylation was confirmed by treating it with Lambda protein phosphatase (Lambda PPase). The addition of Lambda PPase facilitated the release of phosphate groups from the phosphorylated serine, threonine, and tyrosine residues of the ILK5 protein. (c) Mutation of ILK5 on Ser192 residue leads to reduced phosphorylation by P2K1 *in vitro*. Purified GST, GST-P2K1-CD or GST-P2K2-CD recombinant proteins were incubated with ILK5 ^WT^-His or ILK5^S192A^-His, followed by an *in vitro* kinase assay. Auto- and trans-phosphorylation were detected by incorporation of γ-[^32^P]-ATP. MBP and GST-LYK5-CD were used as a universal substrate and a negative control, respectively. Protein loading was visualized using Coomassie brilliant blue (CBB) staining to assess the relative protein amounts. (d) Quantitative analysis was performed to determine the levels of phosphorylated ILK5^WT^ and ILK5^S192A^ protein. The phosphorylation signals of ILK5^WT^ and ILK5^S192A^ by GST-P2K1-CD or GST-P2K2-CD were quantified using Image J and analyzed with GraphPad Prism 8 software. (Supplemental Figure S2). Data shown as mean ± SEM, *n* = 3, **p<0.01, *p*-value indicates significance relative to the band intensity of ILK5^WT^-His and was determined by unpaired two-tailed Student’s *t*-test.

## Supplementary Material

Supplemental Material

## Data Availability

The authors declare that all other data supporting the findings of this study are available within the manuscript and its supplementary files or are available from the corresponding author on request.
