# Supplementary material for: Phosphorylation-mediated regulation of integrin-linked kinase 5 by purinoreceptor P2K2
Source: Plant Signal Behav. 2023 Dec 17;18(1):2261743. doi: 10.1080/15592324.2023.2261743 (PMC10730134; doi:10.1080/15592324.2023.2261743)
Supplement: Supplemental Material [file KPSB_A_2261743_SM5620.zip › Kim et al Supplemental data clean.docx]

**Supplemental materials and methods**

**Plant materials and growth conditions**

In this study, *Arabidopsis thaliana* ecotype Columbia (Col-0) plants were used as a source for protoplasts, while *N. benthamiana* leaves were used for the firefly split-luciferase imaging (LCI) assay. The seeds were surface sterilized by immersion in a 1% (v/v) bleach solution for 10 minutes, after which they were rinsed five times with sterilized water. Seeds were germinated on agar plates using half-strength Murashige and Skoog (MS) medium supplemented with 1% (w/v) sucrose, 0.5% (w/v) phytagel, and 0.05% (w/v) MES. The pH of the medium was adjusted to approximately 5.7 with KOH. Subsequently, the seeds were vernalized at 4 °C in the absence of light for a period of three days. Following vernalization, the plates were transferred to a growth chamber to facilitate germination. During the growth phase, the plants were exposed to long-day conditions, consisting of 16 hours of daylight with a light intensity of 150 *μ*E∙m^-2^∙s^-1^ (where E stands for Einstein, equivalent to 1 mol of photons). The light source used white LED lamps, and the growth temperatures were carefully controlled, maintained at 20 °C for Arabidopsis plants, and 28 °C for *N. benthamiana* plants.

**Protoplast isolation and BiFC assay**

To isolate Arabidopsis protoplasts, 4-week-old plants were subjected to a 30 minute incubation with 1 M mannitol. Following this, 30 mL of a 0.22 μm filter-sterilized enzyme solution was added, which comprised 10 mM MES-KOH (pH 5.7), 0.4 M mannitol, 1 mM CaCl_2_, 1% (w/v) cellulase (Onozuka R-10), 0.25% (w/v) macerozyme (R-10), 1% (w/v) BSA (Goldbio), and 0.035% *β*-mercaptoethanol. After the incubation at 21 °C for 10 hours with gentle agitation, the plates were kept in the dark. Subsequently, the enzyme solution containing protoplasts was carefully filtered through a 75 mm nylon mesh into a 50 mL falcon-tube. The isolated protoplasts were gently layered with 10 mL of W5 solution (2 mM MES pH 5.7, 154 mM NaCl, 125 mM CaCl_2_, and 5 mM KCl) without disturbing the 21% (w/v) sugar solution. Subsequently, centrifugation was carried out for 10 minutes at 100 x g. The intact protoplasts, approximately 10 mL in volume, which floated on top of the sucrose layer, were carefully transferred to a new 50 mL tube, and then an additional 25 mL of W5 solution was added. After that, 15 mL of W5 solution was added to the protoplasts, and centrifugation was performed for 4 minutes at 60 x g. Subsequently, the protoplasts were washed with an additional 15 mL of W5 solution and centrifuged again for 4 minutes at 60 x g repeatedly. The pelleted protoplasts were then resuspended in 5 mL of MaMg solution, which contained 4 mM MES pH 5.7, 0.4 M mannitol, and 15 mM MgCl_2_. In each transformation, approximately 2 × 10^6^ protoplasts were used with 15 *μ*g of each plasmid DNA. The Split-YFP protein fusion plasmids (*pAM-PAT-GW-nYFP* and *pAM-PAT-GW-cYFP*) were introduced into Arabidopsis protoplasts derived from leaf tissues of 4-week-old plants using the polyethylene glycol (PEG)-mediated transformation method, following a previously established procedure.^1^ After resuspension in 2 mL of W5 solution, the protoplasts were incubated in the dark for 24 hours to prepare for subsequent experiments. The YFP fluorescence was monitored using a Zeiss Axiovert 200M inverted Microscope with ORCA-ER camera 24 h after transformation. As a counterstain to visualize the plasma membrane, we utilized 5 *μ*M FM4-64 (Invitrogen, T3166).

**Firefly split-luciferase complementation imaging (LCI) assay**

Full-length cDNAs for each construct were cloned with split-LUC at their C-termini of the *pCAMBIA1300-GW-nLUC* or *pCAMBIA1300-GW-cLUC* vector. Subsequently, *GV3101* cells were transformed by electroporation (BioRad GenePluser^TM^). Once the OD_600_ reached 1.0, the cultures were resuspended in infiltration buffer [containing 10 mM MES (pH 5.7), 10 mM MgCl_2_, and 100 *μ*M 4'-Hydroxy-3',5'-dimethoxyacetophenone] and incubated for 2 hours. Subsequently, *GV3101* agrobacterium carrying the specified constructs (OD_600_ = 0.6) were infiltrated into the leaves of 4-week-old *N. benthamiana* plants. After infiltrating the leaves, they were kept at 28 °C for 3 days before measuring the LUC activity. Subsequently, a solution containing 5 mM D-luciferin and 0.01% (v/v) Triton X-100 was sprayed onto the *N. benthamiana* leaves. The leaves were then placed in darkness for 10 minutes to suppress autofluorescence. The luminescence was recorded and visualized using a low-light imaging CCD camera (Photek; Photek, Ltd.).

***in vitro* kinase assays**

The pET21a vector (Novagen) was used to fuse His-tagged wild-type ILK5 (ILK5^WT^-His) and the Ser192Ala mutation of ILK5 (ILK5^S192A^-His) at the C-terminus. These plasmids were then transformed into Rosetta^TM^ (DE3) competent cells (Novagen). Subsequently, His-tagged proteins were purified using TALON Metal Affinity Resin (Clontech #635502), following the manufacturer's protocol. The N-terminal fusion of the GST-tagged cytosolic domain of P2K1 (GST-P2K1-CD) and the cytosolic domain of LYK5 (GST-LYK5-CD; used as a negative control)^2^ was achieved by cloning them into the pGEX-5X-1 vector (GE Healthcare). The cytosolic domain of P2K2 was cloned into the pET41a vector to generate the GST-P2K2-CD recombinant protein. Additionally, the free-GST protein (GST) was purified using the pGEX-5X-3 vector (GE Healthcare). The plasmids were introduced into Rosetta^TM^ (DE3) competent cells expressing YopH tyrosine phosphatase. Subsequently, GST-tagged proteins were purified using Glutathione Resin (GenScript #L00206) according to the manufacturer's instructions. As a universal substrate, myelin binding protein (MBP) from Sigma (Cat no. M1891-5MG) was utilized. 2 *μ*g of purified GST or GST-tagged protein kinases were mixed with 2 *μ*g of His-tagged ILK5 protein (ILK5-His) as a substrate in a 25 *μ*L reaction buffer. The reaction buffer contained 20 mM Tris-HCl (pH 7.4), 10 mM MgCl_2_, 100 mM NaCl, and 2 mM ATP, with the option of adding 0.2 *μ*L of radioactive [*γ*-32P] ATP. After incubating the mixture for 1 hour at 30 °C, 5 *μ*L of 5× SDS loading buffer was added, and the mixture was incubated in the thermomixer (Eppendorf, 22331Hamburg) at 100 °C for 5 minutes to stop the reaction. Subsequently, each reaction was separated by electrophoresis in 12% (v/v) SDS-PAGE gels. For the reactions containing radioactive [*γ*-32P] ATP, the gel was auto-radiographed using a Typhoon FLA 9000 Phospho-imager (GE Healthcare) for 12 hours. As a loading control, SDS-PAGE gels were stained with Coomassie Brilliant Blue (CBB).

**Supplemental References**

1. Kim DW, Jeon SJ, Hwang SM, Hong JC, Bahk JD. The C3H-type zinc finger protein GDS1/C3H42 is a nuclear-speckle-localized protein that is essential for normal growth and development in Arabidopsis. Plant Sci 2016; 250:141–53.

2. Chen D, Cao Y, Li H, Kim D, Ahsan N, Thelen J, Stacey G. Extracellular ATP elicits DORN1-mediated RBOHD phosphorylation to regulate stomatal aperture. Nat Commun 2017; 8:2265.

**Supplemental Figures**

**
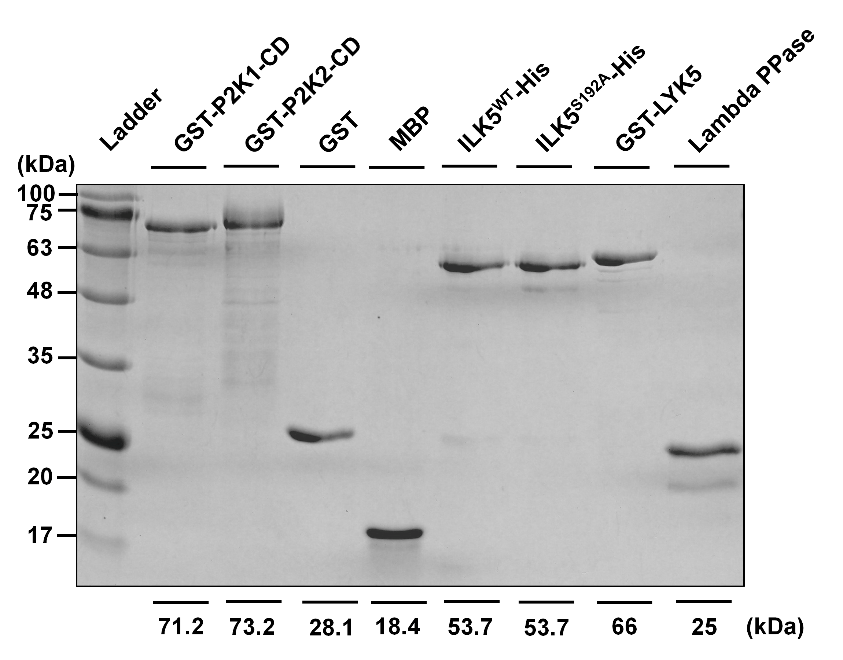
**

**Supplemental Figure S1. Purified recombinant proteins used in *in vitro* kinase**. 2 *μ*g of the following GST-tagged proteins: GST-P2K1-CD (71.2 kDa), GST-P2K2-CD (73.2 kDa), GST-LYK5-CD (66 kDa), and GST (28.1 kDa), were purified using Glutathione affinity chromatography. Additionally, the His-tagged protein, Wild-type and S192A mutated ILK5-His (53.7 kDa), was purified using the IMAC (Immobilized Metal Affinity Chromatography) method. The MBP protein and PPase were obtained from Sigma and NEB (New England Biolabs), respectively. Protein loading was visualized using Coomassie Brilliant Blue (CBB) staining.





**Supplemental Figure S2. Phosphorylation of ILK5^WT^ and ILK5^S192A^ proteins by P2K1 and P2K2 kinase protein**. Purified recombinant ILK5^WT^-His and ILK5^S192A^-His proteins were incubated with GST-P2K1 or GST-P2K2 cytosolic domain (GST-P2K1-CD or GST-P2K2-CD) for *in vitro* kinase assay. Auto- and trans-phosphorylation were detected by incorporation of *γ*-[^32^P]-ATP. The protein loading was visualized by Coomassie Brilliant Blue (CBB) staining. The intensity of each band was measured by autoradiography using ImageJ software (1.52a) and designated in Figure 2D.

**
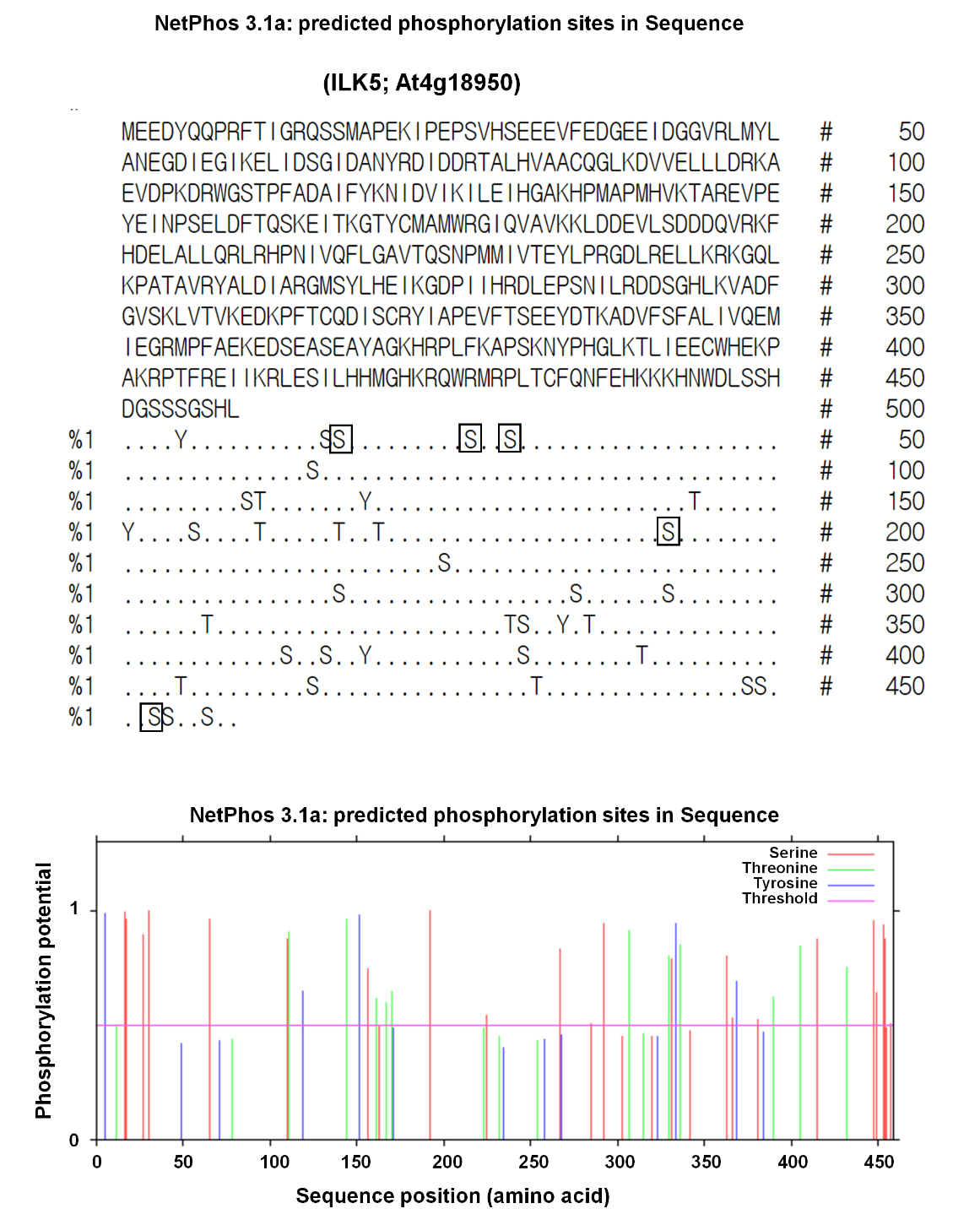
**

**Supplemental Figure S3. Prediction of ILK5 phosphorylation sites using NetPhos 3.1a.** Using the full-length amino acid sequence of ILK5 (as shown in the upper panel), we predicted phosphorylation sites using the NetPhos 3.1a program (https://services.healthtech.dtu.dk/services/NetPhos-3.1/). We have depicted serine, threonine, and tyrosine residues according to their sequence positions (middle and bottom panels). The portion highlighted with a square in the middle panel, including S192, represents five residues (S17, S27, S30, S192 and S453) that have been experimentally confirmed to undergo phosphorylation via Plant PTM viewer (version 2.0; <https://www.psb.ugent.be/webtools/ptm-viewer/index.php>) as shown in Supplemental Table S1.
