# Supplementary figures and images for: Phosphorylation-mediated regulation of integrin-linked kinase 5 by purinoreceptor P2K2
Source: Plant Signal Behav. 2023 Dec 17;18(1):2261743. doi: 10.1080/15592324.2023.2261743 (PMC10730134; doi:10.1080/15592324.2023.2261743)

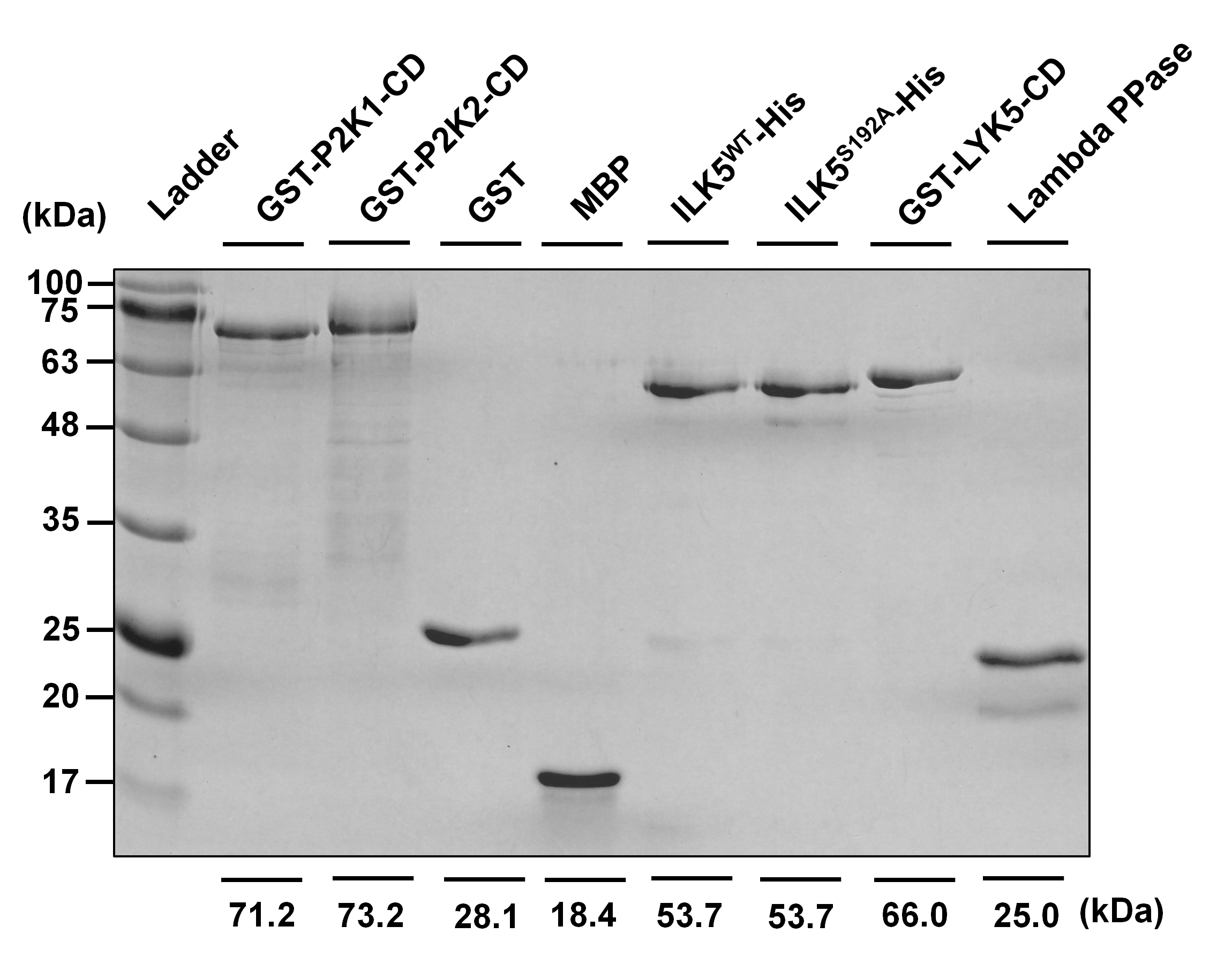

Supplement: Supplemental Material [file KPSB_A_2261743_SM5620.zip › Supplemental Figure S1.tif]

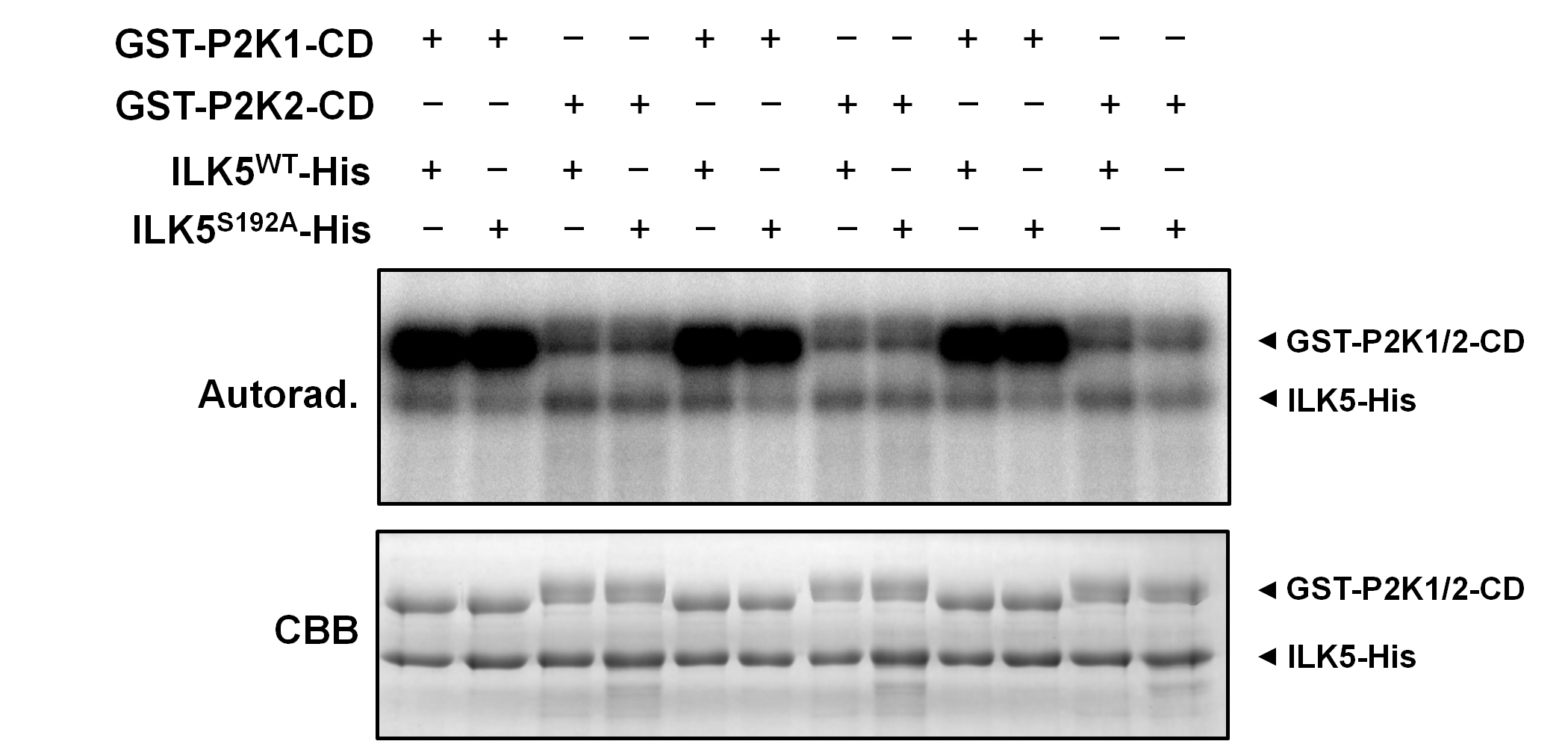

Supplement: Supplemental Material [file KPSB_A_2261743_SM5620.zip › Supplemental Figure S2.tif]

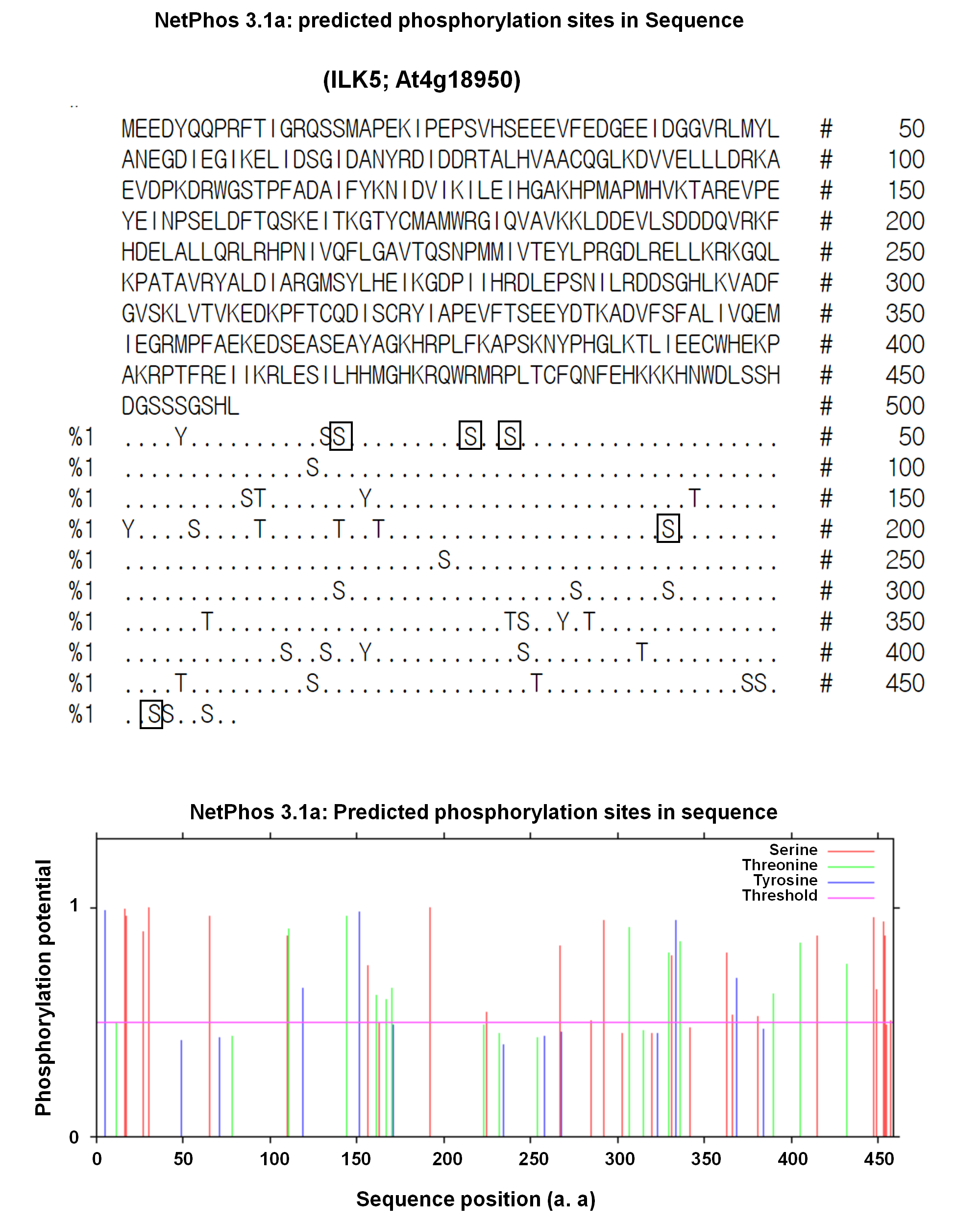

Supplement: Supplemental Material [file KPSB_A_2261743_SM5620.zip › Supplemental Figure S3.tif]
